# High-Frequency Fiber-Optic Ultrasonic Sensor Using Air Micro-Bubble for Imaging of Seismic Physical Models

**DOI:** 10.3390/s16122125

**Published:** 2016-12-14

**Authors:** Tingting Gang, Manli Hu, Qiangzhou Rong, Xueguang Qiao, Lei Liang, Nan Liu, Rongxin Tong, Xiaobo Liu, Ce Bian

**Affiliations:** Physics Department, Northwest University, No. 229, Taibai Road (North), Xi’an 710069, China; tingtinggang1@163.com (T.G.); xgqiao@nwu.edu.cn (X.Q.); lianglei@opt.ac.cn (L.L.); liu_nan07@163.com (N.L.); tong_rongxin@163.com (R.T.); liuxiaobofight@163.com (X.L.); bc459873556@126.com (C.B.)

**Keywords:** Fabry-Perot interferometer, high frequency ultrasonic detection, ultrasonic imaging

## Abstract

A micro-fiber-optic Fabry-Perot interferometer (FPI) is proposed and demonstrated experimentally for ultrasonic imaging of seismic physical models. The device consists of a micro-bubble followed by the end of a single-mode fiber (SMF). The micro-structure is formed by the discharging operation on a short segment of hollow-core fiber (HCF) that is spliced to the SMF. This micro FPI is sensitive to ultrasonic waves (UWs), especially to the high-frequency (up to 10 MHz) UW, thanks to its ultra-thin cavity wall and micro-diameter. A side-band filter technology is employed for the UW interrogation, and then the high signal-to-noise ratio (SNR) UW signal is achieved. Eventually the sensor is used for lateral imaging of the physical model by scanning UW detection and two-dimensional signal reconstruction.

## 1. Introduction

The seismic physical model is a miniature simulation structure according to the proportion of geologic storage structure. As a tool for seismic wave transmission and theoretical predictions, it has several merits such as authenticity of the simulation result, immunity to computing methods and assumed conditions. Compared with the earthquake site, the seismic model laboratory has the characteristics of low cost, good repeatability, stability and reliability. The ultrasonic transducer is the core device taking the inside data of the seismic physical model [1,2,3]. Ultrasonic wave (UW) detection has attracted great interest in applications of biomedical ultrasonography [4], non-destructive testing (NDT) [5] and underwater acoustics and sonar [6] due to its merits of good direction, strong penetration, a long propagation distance in liquid and solid medium. To date, the traditional piezoelectric transducer (PZT) was always the main technique for ultrasonic imaging of physical models [7,8]. However, it has some disadvantages: it is easily disturbed by the ambient electromagnetic field, its large size limits its space resolution, and it has poor multiplex ability [9]. In comparison, the fiber-optic–based sensors have presented outstanding performances for UW detection without the above problems [10]. The side-band filter method is a simple and low-cost technology for responding to high-frequency UWs, and it has been used widely in the UW detection field. The key of this technique is finding a linear spectrum side-band of the sensor. Fiber Bragg grating (FBG) can provide a single-resonance spectrum with a narrow bandwidth, and thus it has attracted great interest in the UW detection field [11,12,13,14]. For further improving the sensitivity, PI (π)-phase delay FBG has a narrower spectral side-band and is usually employed for UW detection instead of FBG. Whenever FBG-based sensors are working, the detected UW frequency is limited by its length (FBG is only sensitive to the UW whose wavelength is shorter than the grating length). In comparison, the fiber-optic Fabry-Perot interferometer (FPI) presents a more compact structure, and thus it acts as a wide-frequency-band detector for high-frequency UW detection [15]. So far, diverse FPI UW sensors have been developed by multiple fiber pretreatment operations, such as etching processing [16], laser micro-machining [17], film coating [18], and hollow-core fiber (HCF) splicing [19]. Although these devices have presented fascinated performances in UW detection, their complex fabrication and poor mechanical strength need be further improved. To achieve ultrasonic detailed imaging of seismic physical models, it is necessary to compress bandwidth in the interference spectrum and improve the signal-to-noise ratio (SNR) of the output signal. More importantly, the size of the sensor should be reduced while still being able accomplish ultrasonic detection with high frequency.

In this paper, we proposed a different FPI with an air micro-bubble cavity for UW detection. The device is formed only by simple HCF-to-SMF (Hollow Core Fiber to Single Mode Fiber) fusion and discharge operation. The sensor presents a compact size of less than 120 μm, and good stability and repeatability, making it a good candidate for scanning the UW imaging of physical models.

## 2. Sensor Fabrication and Operation Principle

**Figure 1:**
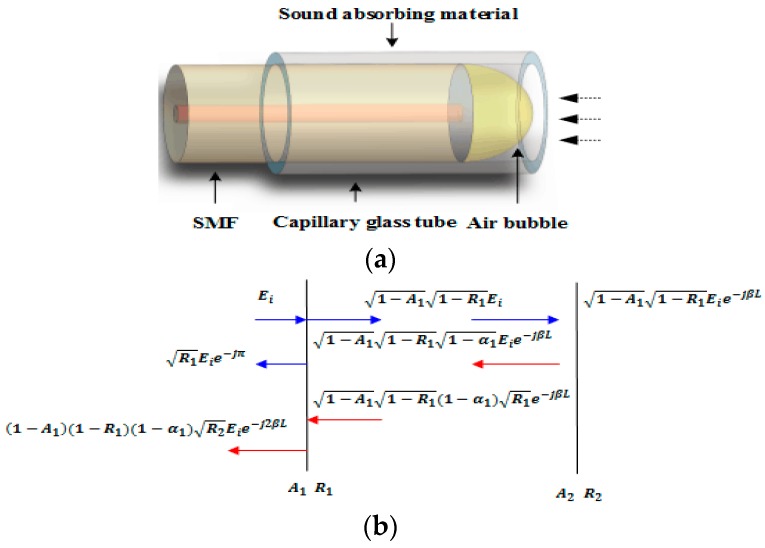

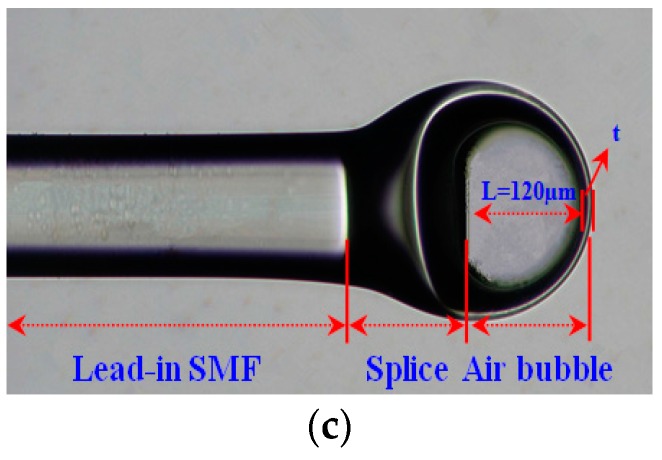
(**a**) Schematic diagram of sensor; (**b**) sensing mechanism; (**c**) photograph of micro-fiber cavity.

The proposed sensor is based on a typical FPI and the detail fabrication process of the micro-bubble is as follows. A 300 µm HCF (the diameters of the hollow core and cladding are 100 µm and 250 µm) is spliced to a leading-in SMF at the discharge condition of−40 bits for power and 4500 ms for time. Then multiple discharge operations are applied to the HCF, and eventually a circular micro-bubble is achieved thanks to the air in the HCF expansion with the discharge induced by the temperature increase. Figure 1a,b show the schematic diagram and the sensing mechanism of the proposed micro-bubble–based FPI, respectively. The FP interference spectrum can be achieved by the double-beam reflected at “1” and “2”. According to Figure 1b [20], the total reflected electric field, $E_{r}$ is shown as:

(1) $$E_{r}\approx \sqrt{R_{1}}E_{i}e^{i\pi }+\left(1-A_{1}\right)\left(1-R_{1}\right)\left(1-\alpha \right)\times \sqrt{R_{2}}E_{i}\exp\left(-j2\beta L\right)$$

where $E_{i}$ is the input field, $A_{1}$ is the transmission loss factor at reflection surface “1”, β is the propagation constant within the air bubble, and α is the loss of the cavity.The length of the micro-bubble is about 120 µm and the wall thickness is 13 µm, as shown in Figure 1c. The sensor is placed into a capillary steel tube with an inside diameter of 0.15 mm and an outside diameter of 1 mm. A certain thickness of the epoxy resin adhesive mixed with tungsten powder is coated on the inside surface of the capillary glass tube to paste the sensor and absorb the residual ultrasonic wave as shown in Figure 1a.

**Figure 2:**
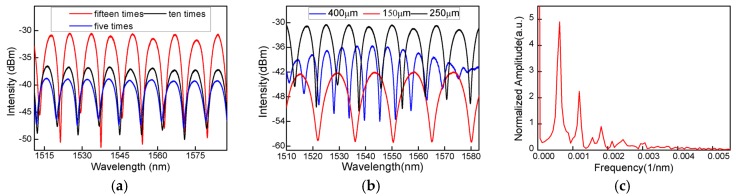
(**a**) Spectrogram in HCF of different discharge times; (**b**) spectrogram at different lengths; (**c**) spatial frequency.

In the process of making the air cavity, the spectrum of the sensor is characterized. It is seen that, as shown in Figure 2a, with increasing the discharge instances, the spectrum presents improved interference patterns with a large fringe contrast. It is because, in the discharge operations, the inside surface of the air cavity becomes smooth, increases the light reflectivity, and eventually results in an expected interference spectrum. In addition, the choice of the length of the HCF is extremely important because the size of the air bubble could be determined by the length of the HCF. Figure 2b shows the reflection spectra of the air bubble made by different lengths of HCF in the wavelength range of 1510–1582 nm under standard atmospheric pressure and room temperature. It can be seen that the free spectral range (FSR) of the spectra has an observable decrease. However, the spectra appear to have a decreased reflected intensity as the length of the HCF change from 250 µm to 400 µm, which is because of the increased wall thickness. Through repeated experiments and comparisons, we chose 250 µm as the length of the HCF owing to the high extinction ratio (EX) and suitable FSR. Thus, a well-defined interference spectrum is obtained by trial and error.

**Figure 3:**
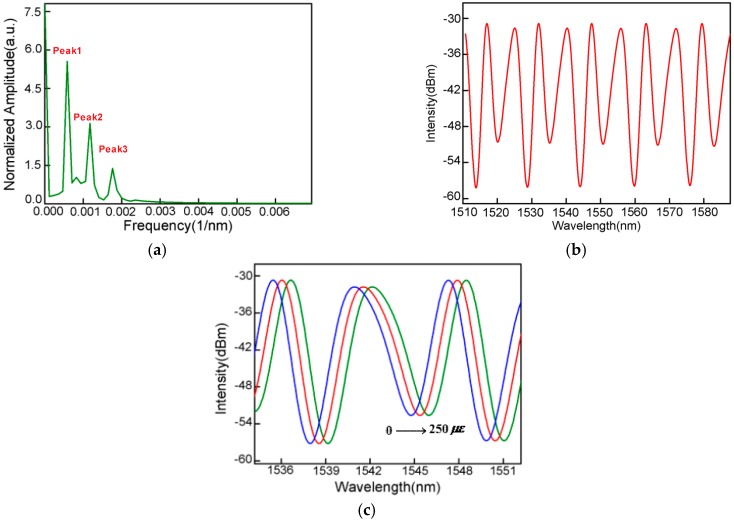
(**a**) Spatial frequency from the simulation result; (**b**) the simulated diagram of the interference pattern; (**c**) reflection spectrum evolution of the air-cavity–based FPI while the tensile strain increases.

To further analyze the characteristics of the interference pattern, the wavelength spectrum has been Fourier-transformed to the spatial frequency, as shown in Figure 2c. It is found that the interference spectrum is a result of not only the interference between the core modes reflected by the end-face of the SMF and the inside surface of the bubble cavity, but also the low-order cladding modes that are generated and participate in the interference. In addition, because of the thin cavity wall, a part of the light may be reflected back to the leading-in SMF by the outside surface of the bubble cavity and then it participates in the interference. This analysis is actually identified by the non-uniform interference spectrum. The interference pattern is inhomogeneous and its simulated diagram is shown in Figure 3b which agrees well with the experimental result. Whenever these weak modulations exist, the large spectral side slope is always achieved for ultrasonic detection.

According to the principle of the multi-wave interferences, the output spectral intensity of the sensor is

(2) $$I=I_{1}+I_{2}+I_{3}-2\sqrt{I_{1}I_{2}}\cos\Delta \varphi_{1}-2\sqrt{I_{2}I_{3}}\cos\Delta \varphi_{2}+2\sqrt{I_{1}I_{3}}\cos\Delta \varphi_{3}\;+I_{1}+I_{2}+I_{3}-2\sqrt{I_{1}I_{2}}\cos\left(\frac{4\pi }{\lambda }n_{air}L+\varphi_{1}\right)-2\sqrt{I_{2}I_{3}}\cos\left(\frac{4\pi }{\lambda }n_{cladding}t+\varphi_{2}\right)\;+2\sqrt{I_{1}I_{3}}\cos\left[\frac{4\pi }{\lambda }\left(n_{air}L+n_{cladding}t\right)+\varphi_{3}\right]$$

where $I_{1},I_{2}$ and $I_{3}$ are the light intensity reflected by the three interfaces, $n_{air}$ and $n_{cladding}$ are the refractive index of the core and cladding, $\varphi_{1}$, $\varphi_{2}$ and $\varphi_{3}$ are the initial phases, *t* is the thickness of the air bubble, and L is the length of the air cavity. The simulated diagram of the proposal sensor is shown in Figure 3b in which t and L are 12 μm and 120 μm. The $n_{air}$ and $n_{cladding}$ are 1 and 1.465, respectively. The FFT (Fast Fourier transform) from the simulation result is shown in Figure 3a. It can be seen that there are two higher-order modes participating in the interference, which cause the slightly negative effect of modifying the main interference spectrum. However, they are too weak to influence the homogeneity of the interference spectrum. Actually, the large effects are attributed to two reasons: the interference between the light reflected by the splicing interface of SMF-to-HCF and the outer wall of the micro-bubble, and the interference between the light of two walls of the micro-bubble. The first interference presents a similar free spectrum range resulting from the similar interference phase difference because of the ultra-thin micro-bubble. As a result, this interference overlaps the main interference spectrum, just broadening the spectral bandwidth. The second interference presents a large FSR because of the ultra-thin micro-bubble as well (which only induces the same phase difference between the reflected light). This interference spectrum also overlaps the main interference spectrum but just plays the function of modifying the interference spectrum. It can be seen that the results agree well with the experimental results.

As the first phase ($\frac{4\pi }{\lambda }n_{air}L+\varphi_{1}$) satisfies an interference valley condition $\left(\varphi =\frac{\pi }{2}\right),$ the corresponding interference minimum wavelength can be expressed as:

(3) $$\lambda_{dip}=\frac{8\pi n_{air}L}{\pi -2\varphi_{1}}$$

The same wavelength shifts are seen between the interference minimum and a point in the spectral side-band chosen in the experiment. According to Equation (3), it can be found that λ is directly proportional to *L*. As the ultrasonic pressure is applied to the FP-based interferometer [21,22,23], it will introduce a strain along the fiber, stretching the air bubble periodically. Thus, the cavity length of the interferometer increases with the ultrasonic pressure application. As a result, the interference spectrum presents a period red-shift, shown in Figure 3c. The phase of the sensor is expressed by

(4) $$\frac{d\lambda }{dp}=\frac{\lambda }{L}\frac{dL}{dP}+\frac{1}{n_{eff}}\frac{dn_{eff}}{dP}$$

The first term depicts the change of the cavity length, and the second term represents the index change. To simplify the discussion, we just state that the length of the sensing fiber can be modulated, which manifests as the modulation of the axial length, and we ignore the elasto-optical effect which can be expressed as

(5) $$\frac{d\lambda }{dp}=\frac{\lambda }{L}\frac{dL}{dP}$$

Actually, the length of the air cavity is the main function of the application of ultrasonic pressure, which can be expressed as

(6) $$\Delta L=\frac{3\left(1-\upsilon^{2}\right)r^{4}}{16Et^{3}}\Delta P$$

where υ is poisson ratio of the wall, *E* is the Young’s modulus of the wall, and *r* is the effective radius of the wall. According to Equations (3) and (4), the acoustic pressure sensitivity of the sensor is derived as

(7) $$\frac{d\lambda }{dp}=\frac{\lambda }{L}\frac{3\left(1-\upsilon^{2}\right)r^{4}}{16Et^{3}}$$

Equation (7) clearly shows that the sensitivity is proportional to the quadruple of the effective radius of the wall, and it is inversely proportional to triple of the thickness of the air bubble. Thus, in the experiment, it is necessary to employ an air bubble with a large radius and thin thickness to improve the sensitivity of the sensor.

**Figure 4:**
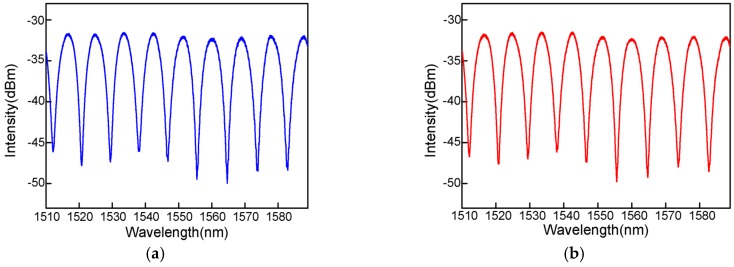
(**a**,**b**) Interference spectrum of the proposed sensor using the same parameters at different time.

Due to the simple fabrication and certain parameters, the proposed sensor can be repeatedly made. Interference spectra of the proposed sensor using the same parameters at different times are shown in Figure 4a,b. It can be found that the two interference spectra are virtually the same.

## 3. Experiment Results

**Figure 5:**
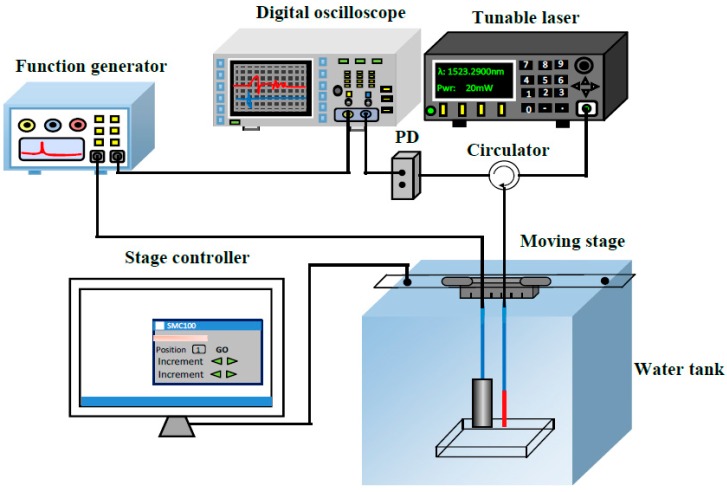
Schematic diagram of fiber-optic ultrasonic detection system.

The schematic diagram of the fiber-optic ultrasonic detection system using the FPI is shown in Figure 5. A spectral filtering technology is used for the ultrasonic interrogation. A light wave with a narrow line width of 0.1 pm and a power of 20 mw has been emitted by a wavelength tunable laser (TSL710, Santec, Aichi Prefecture, Japan) into the FPI and held at a slope of one linear side of the interference spectrum. Its reflection light is transmitted into a PD (Photoelectric detector, 2117-FC-M, New-focus, Trevor, WI, USA) and converted into the electric signal which is shown on an oscilloscope (DS2302A, RIGOL, Beijing, China). A piezoelectric transducer (PZT, mono-crystalline longitudinal wave probe) driven by a function generator with a peak-to-peak voltage of 20 V is used as a source to generate ultrasound waves. Two detected physical models with heights of 5 cm and 3 cm are stacked together and placed in the water.

**Figure 6:**
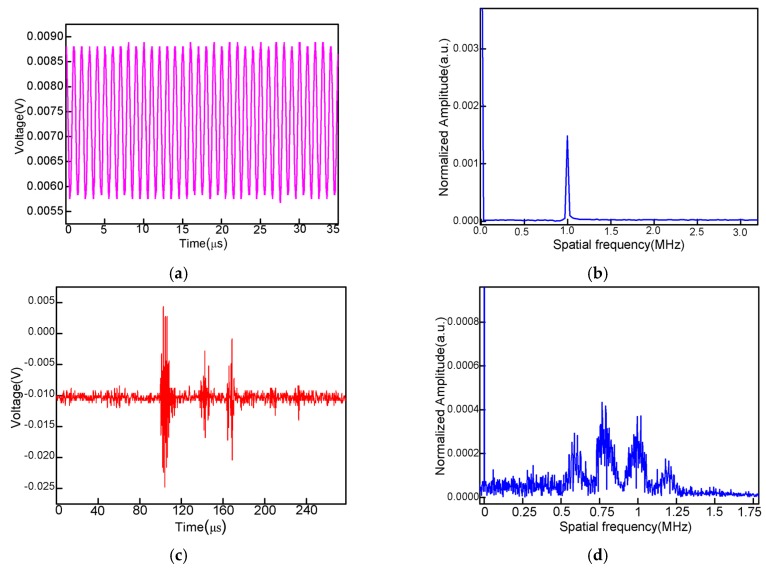
(**a**) Sensor response to a continuous sinusoidal signal reflected by two physical models; (**b**) spatial freqency; (**c**) sensor response to a one-cycle 1MHz ultrasonic pulse; (**d**) spatial frequency;

The sensor’s responses to ultrasounds are characterized as follows. After the PZT and the sensor are held, the sensor can detect the ultrasonic signal reflected by the interfaces of physical models with a high SNR. Figure 6a shows the time domain signal of the continuous ultrasonic signal of 1 MHz. It is transformed to the frequency spectrum, as shown in Figure 6b, and a single frequency is achieved. In order to separate the reflection surfaces of physical models, a pulse ultrasound of 1 MHz is used as the source, and the sensor detects the reflection signals. As shown in Figure 6c, the ultrasonic signals are obtained clearly with respect to three interfaces: the upper surface, the bottom surface, and the bottom surface of the water tank. As the ultrasonic signal propagates to the water–physical model interface, one part of the wave is reflected back to the air bubble, which is received by the PD and converted into an electronic signal; the other part of the wave is refracted and then transmits to the next interface. The ultrasonic velocities in the water and physical model are different (1480 m/s and 2700 m/s, respectively). Theoretically, the travel times of the ultrasonic reflected waves are 98 µs, 141 µs and 168 µs, respectively, which are in agreement with the experimental detecting results shown in Figure 6c. Figure 6d shows the frequency spectrum of the ultrasonic signal in contrast with the frequency spectrum in Figure 6b, which presents a frequency band and several resonant frequencies in the low-frequency band and high-frequency band. These noise signals need to be filtered when imaging the physical model.

**Figure 7:**
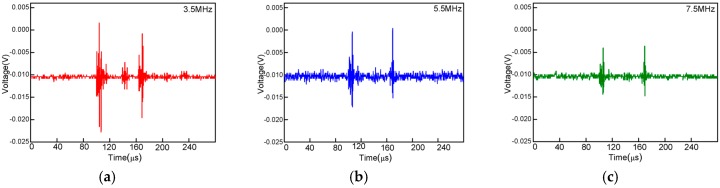
(**a**) Sensor response to one-cycle 1 MHz ultrasonic pulse signals with a frequency of 3.5 MHz; (**b**) 5.5 MHz; (**c**) 7.5 MHz.

Furthermore, the sensor is proposed to measure the high-frequency ultrasounds of 3.5 MHz, 5.5 MHz and 7.5 MHz. Figure 7a–c show the time domain spectra, which prove that the sensor is capable of measuring the ultrasonic wave of a wide frequency band.

**Figure 8:**
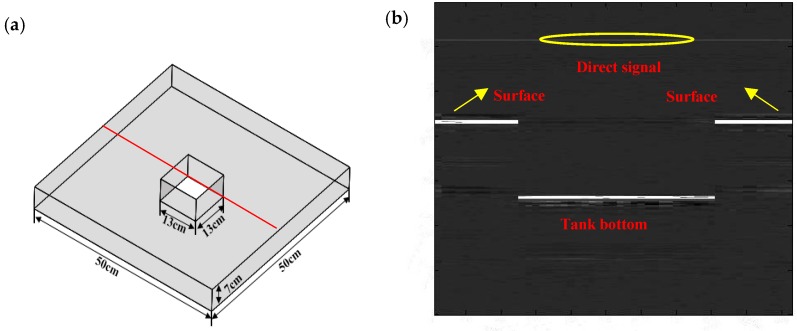
(**a**) Physical models; (**b**) lateral imaging of the physical models.

After the performance study of the sensor probe, the geophysical imaging is demonstrated as follows. The schematic diagram of the ultrasonic imaging of the physical model has been shown in Figure 8a. The model tested is a sunken block with the length and width of 13 cm by13 cm in a larger rectangular Plexiglas block. The model is placed into the water tank. The PZT source and the fiber sensor are held on an electric-driven stage with a spatial resolution of 2 μm for point-to-point scanning. The distance between the PZT and sensor is 3 cm; the water gap between the model and sensor is 5 cm. Figure 8b shows the ultrasonic image of the physical model reconstructed by the time-of-flight approach. As expected, the rectangular pit is clearly performed as expected, in which the edges are clearly separated and detected. In addition, information about the thickness and position of the defect can be obtained through the ultrasonic propagation velocity and time. In this experiment, we employ a band-pass filter near 1MHz for removing its frequency from the surrounding electric machine and the PZT resonance to avoid unexpected noise. Besides, in order to avoid the reflections of other object surfaces, including the bottom and sides of the water tank, sound-absorbing materials are attached to various facets of the water tank. The ultrasound is emitted from the focused transducer in the axial direction which does not cause the observed noise. Even so, there are some noise signals in Figure 8b. On the one hand, the extra noise is mainly attributed to the mode conversion in the physical models. On the other hand, the material of the seismic physical models is absolutely non-uniform, resulting in varying refractions and sounds of speed in the model when ultrasonic signals pass through the seismic physical models. However, the noises are too weak to affect the useful ultrasonic signals.

## 4. Discussion

**Figure 9:**
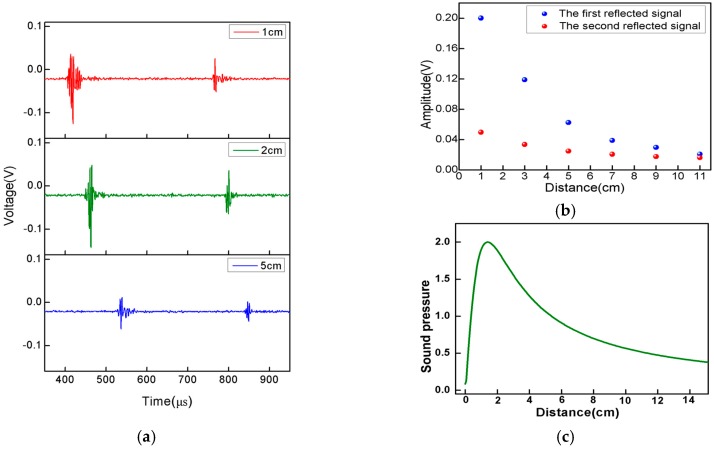
(**a**) Sensor response to a one-cycle 1 MHz ultrasonic pulse with different distances between the sensor and PZT (1 cm, 2 cm, and 5 cm, respectively); (**b**) the amplitude of the reflected signal versus distance; (**c**) the sound pressure distribution of PZT.

In the experiment, the choice of the distance between the sensor and PZT, especially in the ultrasonic imaging section, is vital. Figure 9a shows the sensor response to a one-cycle 1 MHz ultrasonic pulse with a distance between the sensor and PZT of 1 cm, 2 cm, and 5 cm, respectively. The surfaces reflecting the signal of the physical models can be identified in the three graphs. As shown in Figure 9a, it can be seen that although the amplitude of the reflected signal decreases with the increase of the distance, it still can be identified clearly, which means the effective response distance between the PZT and the proposed sensor is above 5 cm under the circumstance of a reflective ultrasonic wave’s detection. In addition, the time delay of the reflected signal increases as the distance gets larger. We plot the peak-to-peak voltages of the signal as the function of the distances between the sensor and PZT as shown in Figure 9b.Additionally, the blue points and red points represent the first and second reflected signals. It can be found that the two reflected signals’ intensity first increases and then gradually decreases as the distance increases. Due to the acoustic-focusing PZT used in the experiment, the distribution of the ultrasonic pressure is complicated in the near field, while in the far field the ultrasonic pressure decreases sharply because of ultrasonic diffusion and transmission loss. As shown in Figure 9c, the sound pressure reaches a maximum when the distance is 2 cm, which is in good agreement with Figure 9b. It is extremely important to set a distance within the linear response area to the ultrasonic pressure (the far field). With continuous experiments and theoretical guidance, the distance between the sensor and PZT was eventually set at 3 cm.

## 5. Conclusions

In this paper, we proposed and demonstrated a micro-bubble–based FPI for high-frequency ultrasound detection. The sensing head is compact with a diameter of 120 µm, and the fabricating process is simple and cost-efficient and only requires a fusion splicer. The experimental results show that the proposed sensor exhibits high sensitivity to both continuous and pulsed ultrasounds of 1 MHz. In addition, the sensor has been implemented for imaging seismic physical models, and the result indicates that the image is clear and has a high resolution. Considering its miniature structure, the proposed sensor is a good candidate for ultrasonic detection and ultrasonic imaging in narrow spaces.
